# Vendor-based restrictions on pesticide sales to prevent pesticide self-poisoning - a pilot study

**DOI:** 10.1186/s12889-018-5178-2

**Published:** 2018-02-20

**Authors:** Manjula Weerasinghe, Flemming Konradsen, Michael Eddleston, Melissa Pearson, Shaluka Jayamanne, David Gunnell, Keith Hawton, Suneth Agampodi

**Affiliations:** 1 Department of Community Medicine, Faculty of Medicine & Allied Sciences, Rajarata University of Sri Lanka, Anuradhapura, Sri Lanka; 2 South Asian Clinical Toxicology Research Collaboration, Faculty of Medicine, University of Peradeniya, Peradeniya, Sri Lanka; 3 Centre for Pesticide Suicide Prevention, and Pharmacology, Toxicology and Therapeutics, Centre for Cardiovascular Science, University of Edinburgh, Edinburgh, UK; 4 Department of Public Health, Faculty of Health and Medical Sciences, University of Copenhagen, Copenhagen, Denmark; 5 Department of Medicine, Faculty of Medicine, University of Kelaniya, Ragama, Sri Lanka; 6 Population Health Sciences, University of Bristol, Bristol, UK; 7 Centre for Suicide Research, Department of Psychiatry, University of Oxford, Oxford, UK

**Keywords:** Pesticides, Pilot study, Pesticide shops, Self-poisoning, Suicide, Sri Lanka

## Abstract

**Background:**

In South Asia, up to 20% of people ingesting pesticides for self-poisoning purchase the pesticide from a shop with the sole intention of self-harm. Individuals who are intoxicated with alcohol and/or non-farmers represent 72% of such high-risk individuals. We aimed to test the feasibility and acceptability of vendor-based restrictions on pesticide sales for such high-risk individuals.

**Methods:**

We conducted a pilot study in 14 (rural = 7, urban = 7) pesticide shops in Anuradhapura District of Sri Lanka. A two-hour training program was delivered to 28 pesticide vendors; the aim of the training was to help vendors recognize and respond to customers at high risk of pesticide self-poisoning. Knowledge and attitudes of vendors towards preventing access to pesticides for self-poisoning at baseline and in a three month follow-up was evaluated by questionnaire. Vendors were interviewed to explore the practice skills taught in the training and their assessment of the program.

**Results:**

The scores of knowledge and attitudes of the vendors significantly increased by 23% (95% CI 15%–32%, *p* < 0.001) and by 16% (95% CI 9%–23%, p < 0.001) respectively in the follow-up. Fifteen (60%) vendors reported refusing sell pesticides to a high-risk person (non-farmer or intoxicated person) in the follow-up compared to three (12%) at baseline. Vendors reported that they were aware from community feedback that they had prevented at least seven suicide attempts. On four identified occasions, vendors in urban shops had been unable to recognize the self-harming intention of customers who then ingested the pesticide. Only 2 (8%) vendors were dissatisfied with the training and 23 (92%) said they would recommend it to other vendors.

**Conclusions:**

Our study suggests that vendor-based sales restriction in regions with high rates of self-poisoning has the potential to reduce access to pesticides for self-poisoning. A large-scale study of the effectiveness and sustainability of this approach is needed.

**Electronic supplementary material:**

The online version of this article (10.1186/s12889-018-5178-2) contains supplementary material, which is available to authorized users.

## Background

Pesticide self-poisoning is one of the three most common global means of suicides [1]. It is estimated that worldwide annually between 110,000 and 168,000 deaths are due to pesticide self-poisoning [2]. In addition, non-fatal poisonings with pesticides add a further burden many times higher than fatal attempts [1].

In Sri Lanka, the rate of suicide increased substantially from the early 1960s until the 1980s because of the widespread introduction and use of pesticides among small-scale famers throughout the country [3]. However, since 1995, the suicide rate has declined dramatically, coinciding with bans of the most toxic pesticides [4, 5] Despite this reduction in deaths, pesticide self-poisoning remains the most common method of self-harm in rural Sri Lanka [6].

The regulation of pesticides in Sri Lanka has been mandated through the Control of Pesticide Act 1980 with several subsequent amendments [7]. The Act provides for regulation of the import, formulation, use, sale, packaging, labeling, storage and transport of pesticides. As part of the Act, pesticides can be sold only from authorized pesticide shops. Pesticide vendors are required to participate in a one-day training that covers such issues as labeling, storage, and toxicity of pesticides, and to pass a written license examination to run a pesticide shop. However, the training does not cover recognition and responding to purchasers at risk of suicide.

Pesticides are freely available from pesticide shops [8] and can be easily purchased at moments of crisis. Studies in Sri Lanka show that 14–20% of pesticide self-poisonings followed pesticide purchases from a shop specifically for the act [9, 10]. In a recent study, the incidence of pesticide self-poisoning in rural Sri Lanka estimated as 305.6 per 100,000 population [6]. This reflects an incidence of pesticide purchase for self-poisoning ranging from 42.8 to 61.1 per 100,000. Up to now, no studies have investigated whether interventions implemented through pesticide vendors might be an acceptable means of prevention.

### Development of the intervention

We undertook a series of studies to develop an intervention that might reduce access to pesticide from shops for self-poisoning. First, in a qualitative study with pesticide vendors, we found that vendors acknowledged the difficulty of distinguishing a customer “at risk” from legitimate customers. Importantly, vendors showed a willingness and enthusiasm to help improve their identification of high-risk customers as the purchase and subsequent death/hospitalization caused much social and community distress [8]. Then, in a case-control study we identified two distinguishing risk factors that might be recognizable by a pesticide vendor - being intoxicated and being a non-farmer (Weerasinghe et al., submitted). Individuals who are intoxicated with alcohol and/or non-farmers represent 72% of such high-risk individuals. Finally, this was followed up by study with local stakeholders, which revealed that nearly all local stakeholders supported a focused training for pesticide vendors to encourage restrictions on pesticide sales to customers at high-risk of self-poisoning (Weerasinghe et al., in press).

This study’s aim was to test the feasibility and acceptability of training of pesticide vendors to restrict sales to customers at high-risk of self-poisoning. An effective intervention raises the possibility of saving many lives every year in low and middle income countries (LMIC).

## Methods

### Design

A pilot study was conducted with pesticide vendors. A two-hour training program was delivered to vendors to recognize high-risk customers and suggest strategies on how to respond them. Knowledge and attitudes of vendors toward preventing access to pesticides for self-poisoning before the training and three months after the training were evaluated. A qualitative assessment was carried out to explore the participants practice skills taught in the training and also their assessment of the training.

The protocol for this study was approved by the Ethics Review Committee of the Faculty of Medicine and Allied Sciences, Rajarata University of Sri Lanka in October 2013, with amendments in June 2015.

### Pesticide shops

This study was carried out in an agricultural area located in the North Central Province of Sri Lanka. The pesticide shops were located in three areas: the Rajanganaya Divisional Secretariat (DS) and in two adjacent town areas, Thambuttegama and Nochchiyagama.

Shops that were located in the Rajanganaya DS were relatively rural, small to medium in size, [8] and sometimes seasonal (open only during the agricultural seasons). The costs of the pesticides in these rural shops were relatively high compared with urban shops but they were more convenient for farmers as they were usually located within walking distance from their fields. The shops selected in Thambuttegama and Nochchiyagama were large urban shops. Most of the farmers in the area visit the Thambuttegama Economic Center to sell their agriculture products.

### Recruitment

**Fig. 1:**
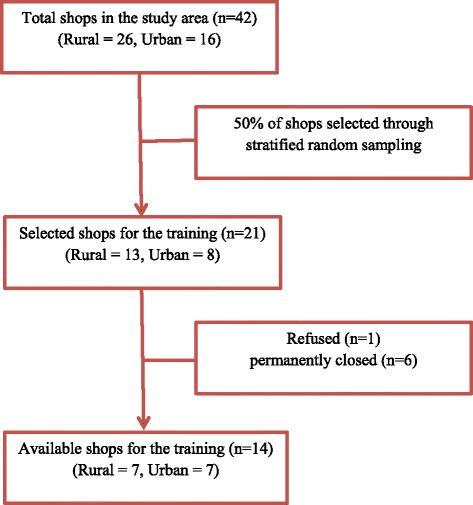
Recruitment of pesticide shops for the study

Project activities were initiated in August 2015. Shops (*n* = 42) that sold pesticides in the study area were identified and mapped as part of a previous study [11]. These pre-identified shops were divided into two strata (rural and urban) based on their location and each shop was assigned unique number. Half of the shops (*n* = 21) from each stratum (50% of urban and 50% of rural) were selected using a random sampling technique. However, among 21 selected shops, 6 shops had permanently closed before starting of the study. Of 15 available shops, 1 refused to take part and remaining 14 (93%) agreed to participate (Fig. 1). Shop staff who directly interacted with customers were invited to the training; staff who did not interact with customers, such as cashiers, were not included. For the purpose of this study, pesticide vendors were defined as shop owners, sales persons (those who giving advice regarding the purchase) or counter assistants (those who passing money to cashier and issuing pesticides) who was involved in the sale of pesticides. Twenty-eight vendors were selected and agreed to participate in the study.

### Intervention

The suicide intervention program developed for medical residents by Kato et al. [12] was used as a model for the development of the current vendor training program. The program was modified to suit the local context where pesticide vendors have limited knowledge of suicide prevention. The training consisted of three different sessions delivered over 2-h (Table 1). The first session was an informal discussion with vendors to allow them to share their previous experiences with high-risk customers. This was followed by a one-hour interactive lecture and was designed to improve the vendors’ knowledge of 1) suicide, pesticide poisoning and prevention; 2) identification of high-risk customers; and 3) employing response strategies. In the second session, vendors were trained to observe any unusual behavior [8] of the customer during the purchase. It was stressed that non-farmers and persons intoxicated with alcohol during the purchase may represent high risk groups for purchasing pesticides for self-poisoning. The vendors were trained to talk with the customers to check their farming credentials and to respond appropriately to high-risk customers. The third session included a 30-min role-play, which was specifically designed to practice skills developed on how to identify and respond to likely high-risk customers.

**Table 1:** Two-hour Training Program for vendors of pesticides to identify and respond to high-risk customers in Sri Lanka

Session 1: Discussion (30 min)	
An informal discussion with vendors to allow them to share their experiences of high-risk customers Expected outcome: Convince vendors that they are in a “strong” position to prevent suicide	
Session 2: Lecture using PowerPoint presentation (60 min)	
(2A). Lecture on suicide/pesticide poisoning as a major public health problem (10 min) Objective: To improve knowledge on suicide, pesticide poisoning and prevention Contents: - Suicide, especially pesticide poisoning, is one of the major public health problems - Every one in five individuals who use pesticides for self-poisoning purchase them from shops - Impulsiveness plays a critical role - Majority of suicides are preventable (2B). Lecture on high-risk customers (30 min) Objective: To improve knowledge on how to identify high-risk customers Contents: 1. Observation of unusual customer behavior (Examples: sadness, nervousness, disheveled appearance, aggressiveness, garbled speech, trembling). 2. Characteristics and unusual behavior of high-risk customers 3. Common ways of asking for pesticides for self-poisoning 4. Common strategies used by high-risk customers to mislead the vendors 5. Most important two risk factors of high-risk customers: (i). Not being a farmer (ii). Intoxication from alcohol during the purchase 6. Common questions that could be asked from the customer to confirm farming status Examples: *“Can you describe the nature of the pest attack?” “What was pesticide you applied the last time?” “What is the age of the crop?”* (2C). Lecture on how vendors can respond to high-risk customers (20 min) Objective: To train vendors on some of the appropriate response to high-risk customers Examples: come back later, return with another family member, contact family member	
Session 3: Role play (30 min)	
Objective: To practice how to identify and respond to high-risk customers (3A). Demonstration (10 min) A short demonstration was performed by the research staff playing roles as a high-risk client with two types of pesticide vendors, including one vendor carefully observing the customer’s behavior, questioning the customer about the purchase and responding to the at risk customer, while the other vendor paid less attention to his customer and sold pesticides without asking further questions. (3B). Practical session (10 min.) A practical session was conducted for participants to practice some of the tips they had learned during the training. Research staff had asked pesticides from vendors in different ways to practice how normally high-risk clients would ask for pesticides and then for the vendors to practice some of the appropriate ways to respond, e.g. come back the following day, bring a family member etc. (3C). Summary and evaluation (10 min.) A feedback for the practical session was provided while summarizing the important points.	

The training was delivered by the principal researcher (MW) with the support of two research assistants. Training was performed in each shop as a time chosen by the vendors. The training was piloted outside of the study area in four pesticide shops (2 rural and 2 urban) and its content updated prior to it being implemented in the study area.

**Fig. 2:**
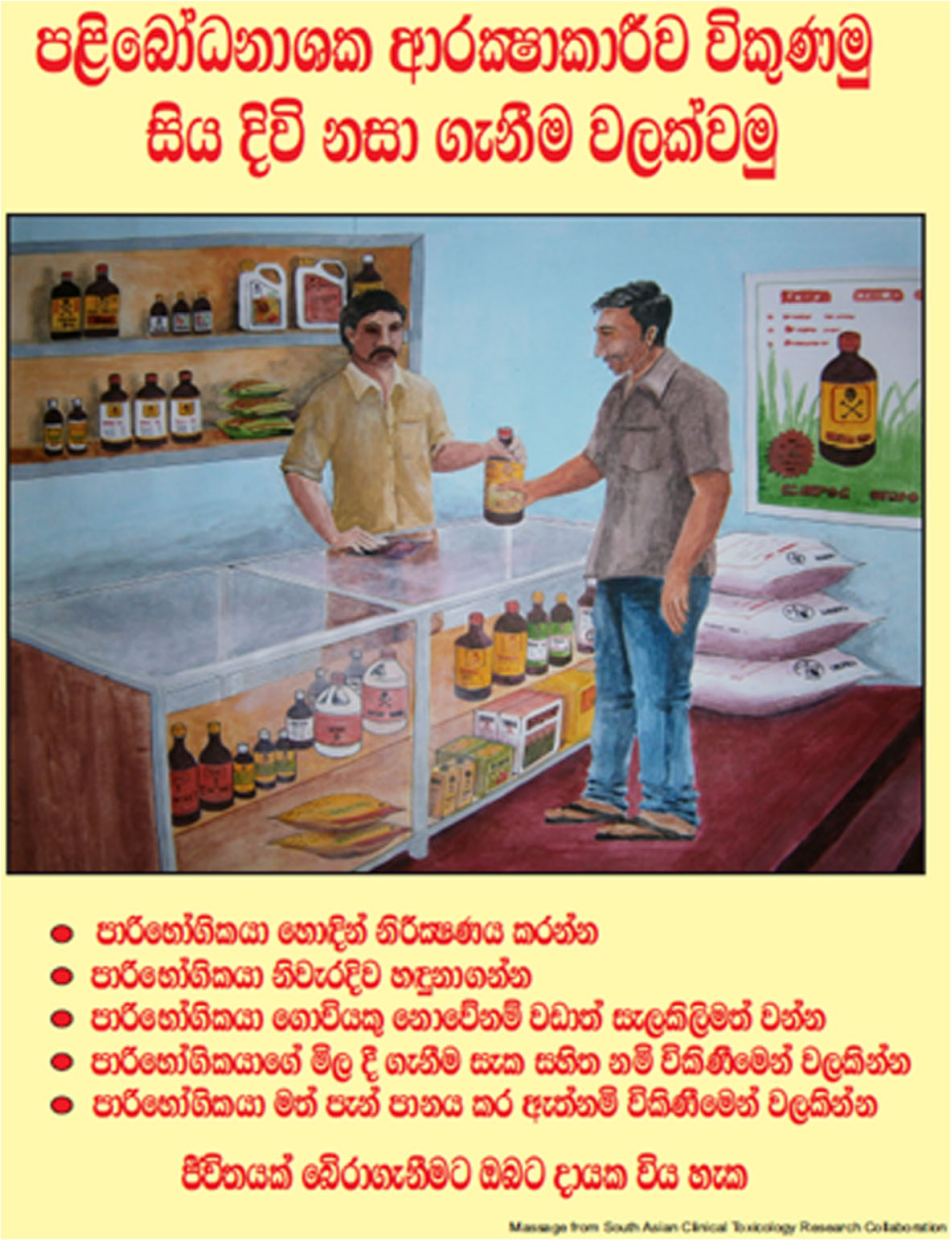
A reminder poster distributed among vendors. It reminds to vendors to make observations, identify customers’ intention, check farming status, not to sell suspicious customers and not to sell alcohol intoxicated customers

Vendors who successfully completed the training were issued with a participation certificate by the local agricultural authority. An additional certificate stating that staff of the shop had undergone a specific training on safe selling of pesticides was issued to each participating shop. A poster was designed and displayed in each shop as a reminder to vendors to make observations, ask questions and respond appropriately to high-risk customers (Fig. 2). A T-shirt with the logo “Safer selling of pesticides” was distributed among participants as a motivational aid.

### Baseline and follow-up surveys

The selected shops were visited by a member of the research team, who explained to the shop staff what the project was about and inviting them to participate. An information sheet setting out further details of the project was provided. It was made clear to the participants that they could opt out of the study at any time.

In both baseline and follow-up surveys, participants were asked to complete self-administered questionnaire (see Additional file 1: Appendix 1), which took approximately 20–30 min to complete. The scale used by Wyman et al. [13] in their gatekeeper training was modified and used to measure vendors’ knowledge, attitudes and practices before and after training. The participants completed the questionnaire individually, but the research team provided support to respondents who needed help in understanding the questions or scales. Three months following the completion of the training (follow-up), course participants completed a second questionnaire similar to the one completed at baseline.

### Follow-up interviews

Interviews were carried out with all twenty-five vendors in their shops to explore their experiences with high-risk customers three months after the training. Each interview was last approximately 30 min. Interviews were audio recorded and then transcribed into local language (Sinhala). Collected information focused on participants practices on the use of questioning, refusals to sell pesticides including reasons for refusals and customer responses, and any knowledge of customers who went on to self-harm. Finally, vendors were asked for their feedback on the training program.

### Data analysis

The questionnaire data were entered into Epi Info version 7. Summary scores for knowledge and attitudes were summed and a breakdown of scoring is presented in Additional file 1: Appendix 1. Paired t-tests were used to determine changes in knowledge and attitude of the vendors after the intervention. Thematic analysis was performed on the qualitative data to examine and record patterns within the data [14, 15]. In keeping with good practice for thematic analysis the principal researcher (MW) the following steps; familiarization by reading through the transcripts, cording each transcript manually identifying main themes and outliers specifically focus on examples on practices and finally refining the codes into the main themes.

## Results

### Characteristics of the pesticide shops

Half of the pesticide shops (7/14, 50%) were rural and small to medium in size. Approximately three-quarters of shops (11/14, 78%) included two or more sales staff. In half of the shops (7/14, 50%) the vendor reported that at least one individual had purchased pesticides for self-poisoning in the past year.

### Vendor characteristics

The median age of the participants was 41 years (IQR = 19). Twenty one of the vendors (21/28, 71%) were males. The participants were from three categories: twelve shop owners (12/28, 43%), thirteen sales staff (13/28, 46%), and three counter assistants (3/28, 11%). Vendors’ experience of selling pesticides was as follows: seven in less than 1 year (7/28, 25%), eleven in 1–5 years (11/28, 39%), ten in over 6 years (10/28, 36%). In all shops, at least one person had undergone training in the compulsory training program conducted by the government. Fourteen participants (14/28, 50%) had not participated in the compulsory vendor training programs. None had previously participated in any training program related to sales restrictions to customers at high risk of suicide.

### Outcome measures

Of the 28 trained vendors, 25 (89%) were available for both the baseline and follow-up assessments and the remaining 3 vendors were not available for follow-up interviews. The scores of knowledge and attitudes of the vendors significantly increased at the follow-up. Knowledge score increased by 23% (95% CI 15%–32%, *p* < 0.001) and attitudes increased by 16% (95% CI: 9%–23%, *p* < 0.001) (see Table 2).

**Table 2:** Knowledge and attitudes of vendors- before and after training

	Knowledge			Attitudes		
	Baseline	Follow-up	% increase (95% CI)	Baseline	Follow-up	% increase (95% CI)
Average	7.84	9.48	23 (15–32)	40.24	46.12	16 (9–23)
*p* value	< 0.001			< 0.001		

### High-risk customers

At baseline, three (3/28, 11%) vendors reported four incidences of refusing to sell pesticide during the previous three months on the basis of the customer’s behavior indicating s/he was considering self-poisoning. Some of the reasons put forward by the vendors for refusal were customers’ young age and unusual appearance, e.g. being disheveled. By contrast, at the follow-up, fifteen (15/25, 60%) vendors reported that they had refused to sell pesticides to 21 individuals (due to the customers possibly considering self-poisoning, being under age or not knowing which pesticide they wanted to buy). The remaining 10 vendors reported that they did not come across any suspicious customers.

Twenty one (21/25, 84%) vendors reported that the training had increased their awareness of not sell pesticides to alcohol-intoxicated customers, while the reports of the remaining four (4/25, 16%) vendors indicated that they remained at the same level after the training. Most of the twenty one incidents where vendors had refused to sell pesticides were because the customers (all males) were under the influence of alcohol at the time of purchase. Examples included:

*“A drunk man came and told me that the vegetable price had dropped and asked for a pesticide. But I did not give him”-* (male vendor, urban shop).

*“A man came after getting drunk … he was about 30 years and asked for Marshal 20. He does not know much about pesticides, so I did not give it to him”-* (male shop owner, urban shop).

Twenty (20/25, 80%) vendors reported that following the training they had questioned more customers to check their farming experiences. Despite the training, five (5/25, 20%) vendors did not think they had changed their behavior on checking customers’ farming experiences.

The rural vendors reported that the majority of their customers were from the same community and were known to them personally, whereas urban vendors reported that most of their customers were from surrounding villages and were not known to them. If the customer was unknown, vendors had made attempts to check their farming experience by asking a few questions before selling the pesticides (see examples in Table 1), as presented by a male shop owner in an urban area:

*“A guy of 20 years came. Firstly, he asked for weedicides. When I asked questions, he got confused. Then he asked for a small weedicide bottle….”*

Another male vendor from an urban shop explained his experience with high-risk customers as follows:

*“A woman of 40-42 came to ask for an insecticide for chilli plants. When we asked her some questions, she failed to respond. It made her suspicious and she left”.*

The same vendor explained another experience with a young boy who had attempted to purchase pesticides:

“*A school boy in his uniform came and asked for an insecticide. I asked for his age (16 years) and told him to look at that training certificate you gave us, asking him to come with a parent since he was underage”.*

This vendor explained that sometimes farmers send their children to buy pesticides on their behalf but that it was better to take precautions when selling pesticides to children. He further explained if a child came with a prescription or note from their parents he would not be suspicious.

### Prevented self-poisoning attempts

In the follow-up survey, vendors reported that over the 3-month period that they were made aware by the community that they had prevented at least seven suicide attempts (five by men). Out of the seven prevented cases reported, three were from rural shops.

*“That guy (55 years old, a mason) does not buy pesticides very often. He had some problems at home. Also he was drunk at that moment. He asked for a gum to admix with pesticides and to spray. I immediately recognized his intention, I told him that there is no such pesticide. I talked a lot with him and changed his mind. I made his home aware of this matter”-* (male shop owner, rural shop).

A few vendors either actively responded to high-risk customers or followed up in order to prevent them seeking alternative means of self-harm. Examples include:

*“A young boy came and asked for a pesticide. He was acting suspiciously and we gave him liquid fertilizer. We did this to prevent him from going to another shop. Since the fertilizer is not that poisonous, it is okay to give that kind of stuff when we are not sure about the customer. He was like 25-30”-* (male sales person, urban shop).

*“A boy of 25 years came restlessly asked for any pesticide. Then we talked to him and realized that he was asking it not for farming. Then we gave some pesticide powder and we went to his place. There was a fight and that powder packet was snatched”-* (female vendor, rural shop).

In addition to the seven prevented cases, two high-risk individuals were turned away and subsequently accessed pesticides from another shop where the vendor had not been trained. A male vendor from a rural shop explained his experience as follows:

*“A person of 29 years came and hassled me by asking for poison. I did not give it to him. Later on I heard that had had bought a pesticide from another shop, drunk it and died”.*

### Unrecognized self-poisoning attempts

Urban vendors reported that over the follow-up period they were aware from community feedback of two occasions they had been unable to recognize the real intention of the customers and who had subsequently ingested pesticide. These vendors reported that they had not identified their intention because these high-risk customers had visited the shop during very busy hours.

### Trainee satisfaction and feedback on the training program

Sixteen (16/25, 64%) of the vendors were fully or fairly satisfied with the training program, while two (2/25, 8%) were fairly dissatisfied; the others (28%) were neutral. The majority of the trainees provided positive feedback on the content, distributed materials, usefulness of the training experience and time allocation. Of note, twenty three (23/25, 92%) said they would recommend the training program to other pesticide vendors. The main reason for dissatisfaction resulted from training taking place during a busy agricultural period and shop time and training taking all the staff away from the shop floor, which resulted in reduced profits.

Some suggestions made by the trainees to improve the training programs including: 1) repeating the training regularly; 2) conducting parallel community awareness programs; and 3) conducting training in small groups to facilitate sharing of experiences and interaction between participants.

## Discussion

We believe that this is the first study evaluating a potential intervention that might reduce access to pesticides from shops for self-poisoning in LMIC. This study provides evidence regarding the possible impact of vendor-based restrictions on pesticide sales as a means of preventing pesticide self-poisoning. It also shows that such an intervention appears to be largely acceptable to vendors.

### Theoretical basis of the intervention

**Fig. 3:**
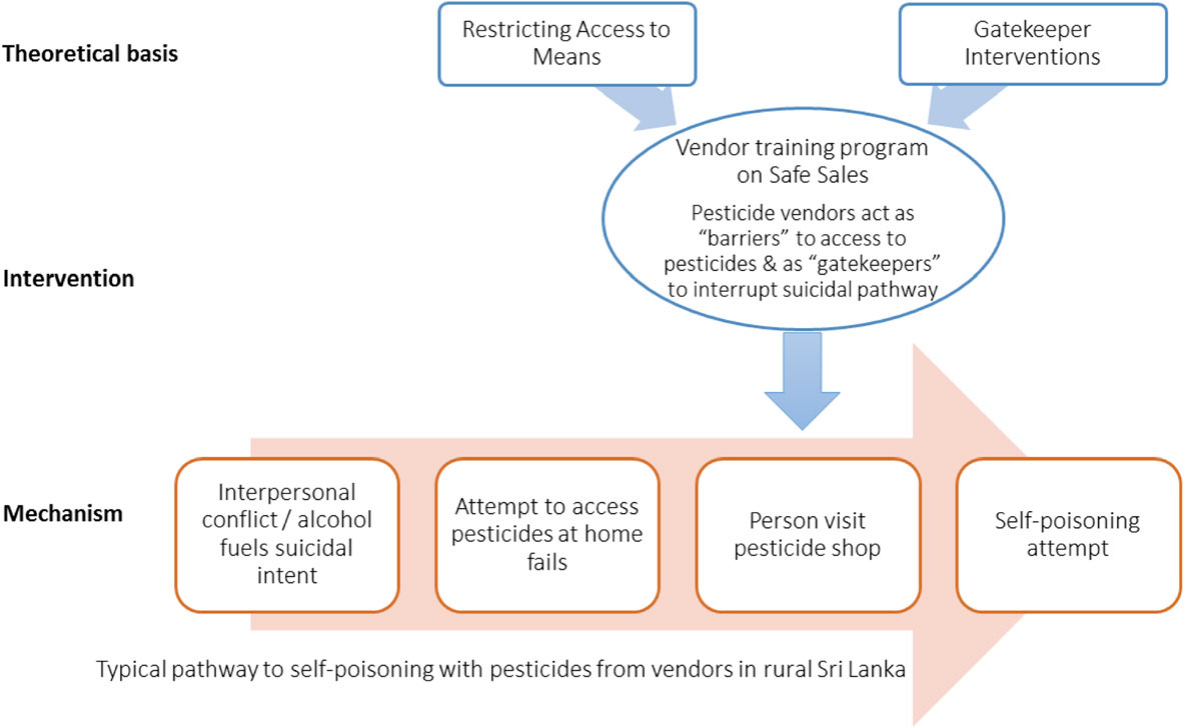
Theoretical model of vendor-based sales restriction

The principle theory behind the current intervention is that a trained vendor can act as a “barrier” by limiting access to pesticides and also as a “gatekeeper” by identifying and responding to high-risk individuals detected amongst legitimate customers (Fig. 3). There is evidence from other suicide prevention research that limiting access to means through sales restrictions and gatekeeper training can be effective. Sales restriction is an approach to limit access to suicide methods and has been successful in a variety of contexts, such as in the UK restrictions on purchasing of analgesic drugs (by limiting the quantity that can be bought in a single purchase) [16, 17] and similarly for caffeine tablets in Sweden [18], barriers to purchase of charcoal in Hong Kong [19], and restrictions on gun sales in many countries [20]. Gatekeeper training has been studied in several populations, including military personnel [21], public school staff [13], peer helpers [22], youth workers [23], clinicians [24], people with depression [25] and indigenous people [26]. This current intervention utilizes these two promising approaches and adapts it to the context of pesticide sales in rural settings.

### The role of vendors in suicide prevention

Pesticide vendors are not professionals (like physicians or trained counselors) or in natural community support roles (such as clergy, and teachers) who may come into contact with suicidal individuals. Therefore, it would not be appropriate to expect pesticide vendors to provide counseling or referrals to other community resources. Instead, due to their contact with potential at high-risk individuals, their willingness to engage, and their membership of the community in which they are located, they are in a unique position within the community to play an active but limited role in prevention of suicide [8].

The participants have prevented seven episodes of pesticide self-poisoning and had refused to sell pesticides to twenty one individuals - much higher than levels prior to training. Vendors’ contact and conversations with people at the point of pesticide purchase provided an opportunity to recognize the intention of the high-risk individuals. Training should involve helping vendors to recognize signs of alcohol intoxication, interview customers to verify their occupation, and build confidence to respond to high-risk customers.

Rural vendors seemed keen to actively engage with customers and showed greater support for the proposed changes than urban vendors. Unfortunately, there was some indication of less engagement of urban pesticide vendors. One possible explanation is that vendors in urban shops found it difficult to check the background of customers, especially during busy shopping hours. Therefore, the proposed vendor training on pesticide sales may be more likely to be effective in rural settings.

### Facilitators and barriers to vendor training

The majority of vendors reported that they were enthusiastic with the intervention. Favorable responses were often reported regarding the training, which encouraged vendors to actively engage in suicide prevention. Further, vendors began taking extra precautions, checking the background of customers and responding in acceptable ways.

There were relatively few barriers to the intervention. These included: 1) vendors’ willingness to take part in the training and change practice, without offering any direct benefits to them; and 2) relatively lower levels of engagement amongst urban vendors.

### Implementation and sustainability

In Sri Lanka, as part of the Control of Pesticide Act, vendors are required to participate in the compulsory training program conducted by the Department of Agriculture [7]. Therefore, training focused on sales restrictions could be relatively easily incorporated into routine training programs, offering the opportunity for a sustained approach. Prior to dissemination to other countries it is important to a) obtain further evidence of impact, on a wider scale, in Sri Lanka, and b) confirm the key factors that would help identify high-risk purchasers, as these may differ in other countries/cultures, such as where alcohol misuse is rare. Further, it may be difficult to implement and sustainability might be questionable depending on the flexibility of pesticide regulations in other settings.

### Limitations of the study

There are several limitations to our study. This study relied on self-reported data from a relatively small number of vendors (*n* = 28 from 14 shops). As such, we cannot reach definitive conclusions about the impact of such training for vendors in reducing fatal and non-fatal self-poisoning. Further, this study was only designed as a pilot study and therefore, included only short-term follow-up that resulted in a limited number of observations and had no control group. One of the major limitations of this study was that it was not designed to assess the feedback of customers who purchased pesticides from trained vendors. Inconveniences may be caused to legitimate customers if they are refused the sale of pesticides and this may impact on the level of community support for such an intervention.

### Future research

The largely positive responses and indications from vendors suggest that this approach to the problem of accessing pesticides from shops for self-poisoning should be further studied for wider implementation. A full-scale evaluation of this strategy would require careful assessment of possible substitution of methods and substitution by shop used for suicidal behavior. It now needs to be formally evaluated in a cluster randomized controlled trial (RCT) before it can be recommended for widespread uptake. This study revealed that high participation rate (93%) of vendors (the vendor who refused declined to himself available and showed little interest to participate the study) which is useful information in power calculation for future RCT.

## Conclusion

This study provides preliminary evidence regarding the possible impact of a vendor-based training on the restriction of the sale of pesticide in the prevention of fatal and non-fatal pesticide self-poisoning. The study suggests that training for all vendors of pesticide in a region has the potential to prevent a substantial proportion of people from purchasing pesticides for self-poisoning, especially in quieter rural shops. Further assessment of the effectiveness of this initiative is needed.

## Additional file

Additional file 1: Appendix 1. Questionnaire for baseline and follow-up survey. (DOCX 40 kb)

## Abbreviations

CI: Confidence Intervals
DS: Divisional Secretariat
IQR: Interquartile Range
LMIC: Low and middle income countries
UK: United Kingdom
RCT: Randomized controlled trial
