# Supplementary material for: Vendor-based restrictions on pesticide sales to prevent pesticide self-poisoning - a pilot study
Source: BMC Public Health. 2018 Feb 20;18:272. doi: 10.1186/s12889-018-5178-2 (PMC5819692; doi:10.1186/s12889-018-5178-2)
Supplement: Supplementary file 1 — Appendix 1. Questionnaire for baseline and follow-up survey. (DOCX 40 kb) [file 12889_2018_5178_MOESM1_ESM.docx]

PILOT STUDY ON VENDOR-BASED RESTRICTIONS ON PESTICIDE SALES TO PREVENT PESTICIDE SELF-POISONING - BASELINE / FOLLOW-UP SURVEY

| Shop ID: | Vendor ID: |
| --- | --- |
| Data Collector initials: | Data collection date: |

*Instructions to the interviewer:*

- *Section I and II need to be complete by the interviewer*

Section I: Shop information

1. Shop location: (check one):

[ ] Urban

[ ] Rural

1. Shop size: (check one):

[ ] Small

[ ] Medium

[ ] Large

Section II: Respondent Profile

1. Age: ____________
2. Gender: [ ] Male [ ] Female
3. Role: *(check one)*

[ ] Owner of a shop

[ ] Sales person

[ ] Counter assistants

[ ] Other (specify) _____________________

1. Experience of selling pesticide in years: *(check one)*

[ ] ≤ 1 year

[ ] 2 - 5

[ ] 6 - 10

[ ] >11

1. Have you ever participate in pesticide “Safe Selling” training?

[ ] Yes

[ ] No

1. In your carrier, how many times have you participate in a training programs related to pesticide sales? Please exclude chemical company promotions. *(check one)*:

[ ] Never

[ ] Once

[ ] Twice

[ ] More than twice

***Instructions to the respondent:***

- *Section III, IV, V and VI need to be complete by the respondent.*
- **Section VI is only relevant to follow up survey.*
- *Please mention first, instinctive answer, even if you don’t think it is “politically correct.” Also not to think about what answers “should” be.*
- *All responses will be coded by an identifying number only and analyzed in group form so that no personal information is revealed.*
- *Some questions may seem similar to each other or redundant, however, answering all questions to help ensure the reliability of the assessment.*
- *Estimated time will be approximately 20-30 minutes to complete this survey.*

**Section III: Knowledge**

1. Please identify whether “true (**✓)**” or “false (X)” following statements

| ***Statements*** | ***Score***  0 = False, 1 = True |
| --- | --- |
| 1. There is a possibility even a familiar farmer to purchase pesticides from your shop with suicidal intention. |  |
| 1. Individuals who purchased pesticides for self-poisoning can be non-farmers |  |
| 1. Most of the individuals who purchased pesticides for self-poisoning are non-farmers |  |
| 1. Individuals who purchased pesticides for self-poisoning can be females |  |
| 1. Individuals who purchased pesticides for self-poisoning can be alcohol intoxicated men. |  |
| 1. Individuals who purchased pesticides for self-poisoning are more likely buy highly toxic but a small pesticide bottle |  |
| 1. Individuals who purchased pesticides for self-poisoning can buy pesticide at any time of the day |  |
| 1. Individuals who purchased pesticides for self-poisoning are always less than 40 years of age |  |
| 1. Questioning and observations are the two methods can used for a vendor to check customers’ purchasing intention |  |
| 1. Reject selling pesticides to non-farmers and alcoholic persons would be the best strategies to prevent access pesticides from vendors for self-poisoning |  |

***(Total score: 0-10)***

**Section IV: Opinions**

For each of the following statements, please indicate your response on the scale from "Strongly Disagree" (1) to "Strongly Agree" (7).

| ***Statements*** | ***Score*** |
| --- | --- |
|  | ***Strongly Disagree Agree Strongly***  ***Disagree Agree*** |
| 1. Prevent pesticide access from shops for self-poisoning is core responsibility of vendors | 1 2 3 4 5 6 7 |
| 1. All customers who come buy pesticides from my shop are legitimate customers. No one purchase pesticides with suicidal intention | 1 2 3 4 5 6 7 |
| 1. I feel comfortable asking questions of any customer to check his/her intention for buying | 1 2 3 4 5 6 7 |
| 1. I have enough skills to recognize a potential suicide risk customer | 1 2 3 4 5 6 7 |
| 1. I feel comfortable in responding to a potential suicidal customer | 1 2 3 4 5 6 7 |
| 1. I have sufficient training in identifying ‘real’ intention of a risk customer and prevent his/her suicidal attempt | 1 2 3 4 5 6 7 |
| 1. I am too busy; so I have no time to check customers’ purchasing intention | 1 2 3 4 5 6 7 |
| 1. I feel guilty if someone accessed pesticides from me and used them for self-poisoning | 1 2 3 4 5 6 7 |

***(Total score: 7-56)***

**Section V: Practice Issues**

1. Have you refused to sell pesticide in the last 3 months thought a customer’s behavior might indicate s/he was considering self-poisoning?

[ ] Yes

[ ] No

1. Have you refused to sell pesticide in the past year thought a customer’s behavior might indicate s/he was considering self-poisoning?

[ ] Yes

[ ] No

1. Did you sell pesticides to customers for self-poisoning without recognizing real intention in the last 3 months?

[ ] Yes

[ ] No

[ ] Not sure

1. How often in the last 3 month have you asked a customer questions to conform a farmer or not?

| *Never* | *Seldom* | Sometimes | Nearly always | Always |
| --- | --- | --- | --- | --- |
| 1 | 2 | 3 | 4 | 5 |

1. How often in the last 3 month have you refused to sell pesticides to a customer who intoxicated alcohol?

| *Never* | *Seldom* | Sometimes | Nearly always | Always |
| --- | --- | --- | --- | --- |
| 1 | 2 | 3 | 4 | 5 |

**Section VI: Training program**

1. Let me know your overall satisfaction about the training program.

| *Fully satisfied* | *Satisfied* | Neutral | Fairly dissatisfied | Strongly dissatisfied |
| --- | --- | --- | --- | --- |
| 1 | 2 | 3 | 4 | 5 |

1. Please let me know whether or not you recommend this training to other pesticide vendors?

[ ] Yes

[ ] No
